# Dietary meat intake and risk of asthma in children: evidence from a meta-analysis

**DOI:** 10.1097/MD.0000000000018235

**Published:** 2020-01-03

**Authors:** Dan Zhang, Lihua Cao, Zhenshan Wang, Zhenqiang Wang

**Affiliations:** Department of Respiratory Medicine, The Second Affiliated Hospital of Dalian Medical University, Dalian 116000, Liaoning, China.

**Keywords:** asthma, children, daily intake, meat intake, meta-analysis, risk factor, 3 or more times

## Abstract

**Background:**

Many studies have been reported that dietary meat intake may be associated with the risk of asthma in children, but the results are inconsistent. Therefore, we performed a meta-analysis to evaluate the effect of meat on the risk of asthma in children.

**Methods:**

The databases PubMed, Embase, and Web of Science were searched. Pooled odds ratios (OR) and 95% confidence intervals (CI) were calculated with random-effect model using Stata software.

**Results:**

A total of 9 articles were included in this meta-analysis. Results from our study suggest that dietary meat intake 3 or more times per week compared with never/occasionally intake has no significant association with asthma risk among children (OR = 1.27, 95% CI = 0.80–2.01, *P* = .308). Similarly, daily intake of meat did not affect the risk of asthma in children when compared with never/occasionally intake (OR = 1.13, 95% CI = 0.93–1.37, *P* = 0.234). In addition, no publication biases were detected in our meta-analysis.

**Conclusion:**

Dietary meat intake most probably is not a risk factor for asthma in children. Due to some limitations that exist in our study, more studies are needed to further assess the association between meat intake and asthma risk in children.

## 1 Introduction

Asthma, as a chronic inflammatory airway disease characterized by bronchial hyperresponsiveness, is currently one of the most common childhood diseases for mandatory chronic drug treatment.^[1]^ As far as we know, in 2017, the World Health Organization had cleared that approximately 235 million people worldwide suffer from asthma, and the sharp rise in asthma prevalence has become a serious public health problem.^[2]^ Asthma in children is rapidly increasing. This disease is complex with a wide range of potential determinants and is associated with protective and deteriorating factors.^[3]^ Diet is one of the factors that influence the development of asthma.^[4]^ Previous study has shown that the Mediterranean diet in children may prevent asthma or wheezing, but randomized controlled trials are lacking.^[5]^ Studies have also shown a number of associations between early dietary intake and subsequent adiposity, as well as asthma.^[4,6]^ Specifically, a fast-food diet, which contained more processed meat and lack of antioxidants, plays an important role in asthma extension.^[7]^ A study performed by Webb et al^[8]^ suggested that meat consumption has become a favorite diet among 18-month-old children. Previous studies have reported the effect of meat intake on the risk of asthma in children.^[9–17]^ However, based on their findings, the current view of the role of meat in asthma in children is not yet clear. Therefore, we performed a meta-analysis to evaluate the effect of meat intake on the risk of asthma in children.

## 2 Materials and methods

### 2.1 Search strategies

This meta-analysis was carried out according to Preferred Reporting Items for Systematic Reviews and Meta-Analyses (PRISMA) Statement.^[18]^ The databases PubMed, Embase, and Web of Science were searched on March 2019. The citation lists of included studies were also examined. Search terms included “meat” AND “asthma” AND “child.” Two reviewers systematically and independently searched for relevant articles. This study did not require approval by an ethics review committee because it is a meta-analysis.

### 2.2 Inclusion and exclusion criteria

The inclusion criteria were as follows:

1. asthma was diagnosed clearly;
2. studies were among children;
3. factors of interest have included meat intake;
4. having available odds ratio (OR) and its 95% confidence intervals (CI) or enough data for calculating them;
5. published in English.

The exclusion criteria were as follows:

1. case reports, conference abstracts, letters, editorials, reviews;
2. overlapping or duplicate studies;
3. irrelevant studies;
4. if study did not clarify the association between dietary meat intake 3 or more times per week compared with never/occasionally intake and asthma risk in children or reported dietary meat intake (yes vs no) and asthma risk, we also excluded it.

### 2.3 Data extraction

The selection of studies was conducted independently by 2 investigators and any discrepancies were resolved by consensus. The useful information listed in Table 1 was extracted from each study. If data were unavailable in an article, we contacted the authors for relevant data.

**Table 1:**
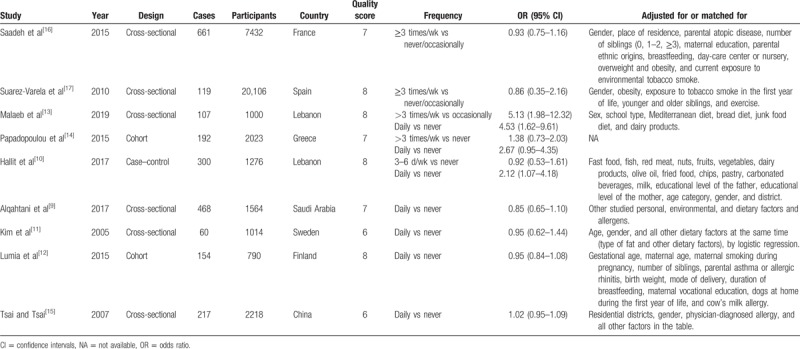
Characteristics of the studies between dietary meat intake and the risk of asthma in children.

### 2.4 Quality assessment

The Newcastle-Ottawa Scale (NOS) was used for evaluating the quality of each study.^[19]^

### 2.5 Search results and study characteristics

**Figure 1:**
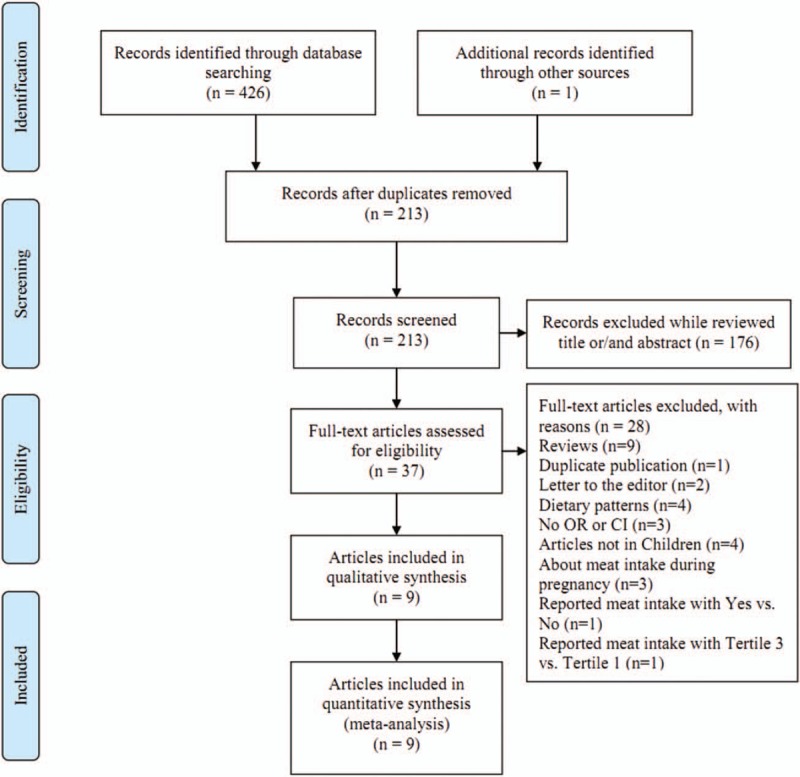
. Flow chart of this meta-analysis.

The flowchart of the literature selection process is shown in Figure 1. The initial retrieval of electronic databases identified 426 records, with 1 additional record identified through the reference of a review; after duplicates from different databases were removed, 213 studies remained. After title and/or abstract examination, 176 papers were excluded and 37 records were evaluated by full-text reading. Twenty-eight full text studies were eliminated because of various reasons (Fig. 1). Finally, 9 articles^[9–17]^ were included in this meta-analysis. All of the 9 studies were of relatively high quality (over 6 stars), with an average NOS score of 7.22. The baseline characteristics of included studies are shown in Table 1.

### 2.6 Statistical analysis

Pooled OR and 95% CI were used to analyze the relationship between meat intake and asthma risk in children.^[20]^ We first calculated the log value of OR and 95% CI in each included study, then combined these ORs.^[20]^ Results in random-effect model have a wider CI and were more cautious than fixed-effect model. Therefore, a random-effect model was used in the pooled analysis.^[21]^ Cochran *Q* test and Higgins *I*^2^ statistic were used to assess the heterogeneity among studies. A *P* < .10 for *Q* test or *I*^2^ > 50% for *I*^2^ test suggested significant heterogeneity.^[22]^ In addition, subgroup analyses by ethnicity and study design were conducted. Publication bias was estimated using Egger tests.^[23]^ All analyses were carried out with the statistical software Stata (version 12.0, StataCorp, College Station, TX). A two-sided *P* < .05 was considered statistically significant.

## 3 Results

### 3.1 Dietary meat intake 3 or more times per week compared with never/occasionally intake

**Figure 2:**
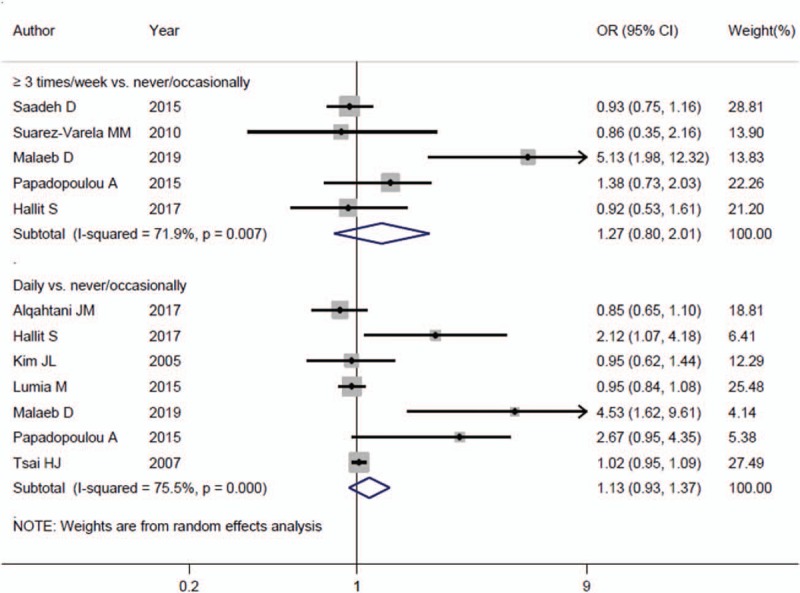
. The forest plot of the association about dietary meat intake 3 or more times per week or daily intake compared with never/occasionally intake on the risk of asthma in children.

Five studies^[10,13,14,16,17]^ were suitable for the analysis in this section. Two of them were from Asia^[10,13]^ and the remaining 3 were from Europe.^[14,16,17]^ Three of them were in cross-sectional design,^[13,16,17]^ 1 in cohort design,^[14]^ and 1 in case-control design.^[10]^ Results from the analysis suggest that dietary meat intake 3 or more times per week compared with never/occasionally intake has no significant association with asthma risk among children (OR = 1.27, 95% CI = 0.80–2.01, *P* = .308; *I*^2^ = 71.9%, *P*_*f*or heterogeneity_ = .007) (Fig. 2). Subgroup analysis by geographic location was performed. The pooled results indicated nonsignificant effect of meat intake on the risk of asthma in children either in Asian populations^[10,13]^ (OR = 2.09, 95% CI = 0.39–11.23) or in European populations^[14,16,17]^ (OR = 0.98, 95% CI = 0.81–1.20). When we conducted the subgroup analysis by study design, the result in cross-sectional studies^[13,16,17]^ (OR = 1.52, 95% CI = 0.56–4.10) was consistent with the overall result.

### 3.2 Daily intake of meat with asthma risk in children

Seven studies^[9–15]^ were published to explore the association about daily intake of meat on the effect of asthma in children. Four of them were from Asia^[9,10,13,15]^ and the remaining 3 were from Europe.^[11,12,14]^ Three of them were in cross-sectional design,^[9,11,13,15]^ 2 in cohort design,^[12,14]^ and 1 in case–control design.^[10]^ As a result, daily intake of meat did not affect the risk of asthma in children when compared with never/occasionally intake (OR = 1.13, 95% CI = 0.93–1.37, *P* = .234; *I*^2^ = 75.5%, *P*_for heterogeneity_ < .001) (Fig. 2). Similarly, the association was nonsignificant in Asian populations^[9,10,13,15]^ (OR = 1.33, 95% CI = 0.89–1.98) or in European populations^[11,12,14]^ (OR = 1.16, 95% CI = 0.76–1.77). When we conducted the subgroup analysis by study design, the results in cross-sectional studies^[9,11,13,15]^ (OR = 1.09, 95% CI = 0.80–1.48) and in cohort studies^[12,14]^ (OR = 1.48, 95% CI = 0.54–4.05) were consistent with the overall result.

### 3.3 Publication bias

Publication bias by Egger tests was not significant in the current meta-analysis in dietary meat intake 3 or more times per week, daily intake compared with never/occasionally intake analysis (*P* = .283 and .149, respectively).

### 3.4 Sensitivity analysis

We conducted sensitivity analysis by sequential omission of individual studies to probe the change in the OR and 95% CI of meta-analysis. As a result, no significant difference was observed when any of the studies was excluded in all correlation assessments, indicating the reliability and stability of the meta-analysis.

## 4 Discussion

In the present study, we comprehensively searched multiple databases and retrieved 11 articles including 2418 cases with regard to the effect of dietary meat intake on the risk of asthma in children. To our knowledge, this study is the first meta-analysis to investigate the role and relevance of meat intake with asthma risk in children. The pooled data showed that dietary meat intake 3 or more times per week or daily intake had no significant association with asthma risk in children when compared with never/occasionally intake. Subgroup analyses by geographic location and study design also did not get a positive result. Taken together, this study indicated that dietary meat intake might be not be associated with the risk of asthma in children.

In our study, we mainly analyzed the association between dietary meat intake 3 or more times per week or daily intake compared with never/occasionally intake and the risk of asthma in children. Three studies (Malaeb et al,^[13]^ Papadopoulou et al,^[14]^ and Hallit et al^[10]^) reported dietary meat intake 3 or more times per week and daily meat intake compared with never/occasionally intake.

Previous studies had suggested that high intake of meat and poultry, and excessive consumption of polyunsaturated fatty acids could contribute to the tremendous increase in asthma prevalence.^[7]^ In our analysis, we failed to get a positive relation between meat intake and asthma in children. Due to the limitation of the data provided in each individual study, we could not distinguish red meat or processed meat. This may affect the subgroup results. Therefore, future studies with detailed information about red meat or processed meat are warranted to explore the further association.

Nevertheless, our study has several limitations. First, significant heterogeneity existed among studies. Meanwhile, subgroup analyses showed that the heterogeneity appeared in some groups. However, no single study had essential effect on the overall OR in a sensitivity analysis. Second, all the included studies come from Asia and Europe. It is unclear whether these findings apply to other populations. Third, most of the included studies were cross-sectional studies. Therefore, further large-scale prospective studies are needed to validate the results.

## 5 Conclusions

In conclusion, our results demonstrate that dietary meat intake most probably is not a risk factor for asthma in children. Due to some limitations that exist in our study, more studies are needed to further assess the association between meat intake and asthma risk in children.

## Author contributions

**Data curation:** Dan Zhang, Lihua Cao, Zhenqiang Wang.

**Investigation:** Dan Zhang, Lihua Cao.

**Methodology:** Dan Zhang, Lihua Cao, Zhenqiang Wang.

**Software:** Lihua Cao, Zhenqiang Wang.

**Writing – original draft:** Dan Zhang.

**Writing – review & editing:** Zhenshan Wang.
